# Heteromeric α/β glycine receptors regulate excitability in parvalbumin‐expressing dorsal horn neurons through phasic and tonic glycinergic inhibition

**DOI:** 10.1113/JP274926

**Published:** 2017-10-19

**Authors:** M. A. Gradwell, K. A. Boyle, R. J. Callister, D. I. Hughes, B. A. Graham

**Affiliations:** ^1^ School of Biomedical Sciences and Pharmacy Faculty of Health University of Newcastle Callaghan NSW Australia; ^2^ Hunter Medical Research Institute (HMRI) New Lambton Heights NSW Australia; ^3^ Institute of Neuroscience Psychology, College of Medical, Veterinary and Life Sciences University of Glasgow Glasgow UK

**Keywords:** pain, glycine receptors, dorsal horn, inhibition, extrasynaptic receptors

## Abstract

**Key points:**

- Spinal parvalbumin‐expressing interneurons have been identified as a critical source of inhibition to regulate sensory thresholds by gating mechanical inputs in the dorsal horn.
- This study assessed the inhibitory regulation of the parvalbumin‐expressing interneurons, showing that synaptic and tonic glycinergic currents dominate, blocking neuronal or glial glycine transporters enhances tonic glycinergic currents, and these manipulations reduce excitability.
- Synaptically released glycine also enhanced tonic glycinergic currents and resulted in decreased parvalbumin‐expressing interneuron excitability.
- Analysis of the glycine receptor properties mediating inhibition of parvalbumin neurons, as well as single channel recordings, indicates that heteromeric α/β subunit‐containing receptors underlie both synaptic and tonic glycinergic currents.
- Our findings indicate that glycinergic inhibition provides critical control of excitability in parvalbumin‐expressing interneurons in the dorsal horn and represents a pharmacological target to manipulate spinal sensory processing.

**Abstract:**

The dorsal horn (DH) of the spinal cord is an important site for modality‐specific processing of sensory information and is essential for contextually relevant sensory experience. Parvalbumin‐expressing inhibitory interneurons (PV+ INs) have functional properties and connectivity that enables them to segregate tactile and nociceptive information. Here we examine inhibitory drive to PV+ INs using targeted patch‐clamp recording in spinal cord slices from adult transgenic mice that express enhanced green fluorescent protein in PV+ INs. Analysis of inhibitory synaptic currents showed glycinergic transmission is the dominant form of phasic inhibition to PV+ INs. In addition, PV+ INs expressed robust glycine‐mediated tonic currents; however, we found no evidence for tonic GABAergic currents. Manipulation of extracellular glycine by blocking either, or both, the glial and neuronal glycine transporters markedly decreased PV+ IN excitability, as assessed by action potential discharge. This decreased excitability was replicated when tonic glycinergic currents were increased by electrically activating glycinergic synapses. Finally, we show that both phasic and tonic forms of glycinergic inhibition are mediated by heteromeric α/β glycine receptors. This differs from GABA_A_ receptors in the dorsal horn, where different receptor stoichiometries underlie phasic and tonic inhibition. Together these data suggest both phasic and tonic glycinergic inhibition regulate the output of PV+ INs and contribute to the processing and segregation of tactile and nociceptive information. The shared stoichiometry for phasic and tonic glycine receptors suggests pharmacology is unlikely to be able to selectively target each form of inhibition in PV+ INs.

## Key points

- Spinal parvalbumin‐expressing interneurons have been identified as a critical source of inhibition to regulate sensory thresholds by gating mechanical inputs in the dorsal horn.
- This study assessed the inhibitory regulation of the parvalbumin‐expressing interneurons, showing that synaptic and tonic glycinergic currents dominate, blocking neuronal or glial glycine transporters enhances tonic glycinergic currents, and these manipulations reduce excitability.
- Synaptically released glycine also enhanced tonic glycinergic currents and resulted in decreased parvalbumin‐expressing interneuron excitability.
- Analysis of the glycine receptor properties mediating inhibition of parvalbumin neurons, as well as single channel recordings, indicates that heteromeric α/β subunit‐containing receptors underlie both synaptic and tonic glycinergic currents.
- Our findings indicate that glycinergic inhibition provides critical control of excitability in parvalbumin‐expressing interneurons in the dorsal horn and represents a pharmacological target to manipulate spinal sensory processing.

## Introduction

The dorsal horn (DH) of the spinal cord contains a heterogeneous population of neurons that process information related to nociceptive, light touch, itch and thermal modalities (Todd, 2010). Integration or segregation of these modalities is considered critical for normal sensory experience, and inappropriate mixing of sensory signals in the DH is thought to cause aberrant sensations such as allodynia, hyperalgesia, spontaneous pain and itch. A large literature, beginning with the gate control theory of pain (Melzack & Wall, 1965), assigns a crucial role for synaptic inhibition in maintaining contextually relevant sensory processing, employing both *in vivo* (Yaksh, 1989; Ishikawa *et al*. 2000) and *in vitro* preparations (Ruscheweyh & Sandkuhler, 2005; Takazawa & MacDermott, 2010). More recently, a series of sophisticated studies employing neuron‐specific transplantation (Braz *et al*. 2012), paired recordings (Lu & Perl, 2003), and targeted ablation, silencing and activation (Duan *et al*. 2014; Bourane *et al*. 2015; Foster *et al*. 2015; Petitjean *et al*. 2015; Cui *et al*. 2016) have confirmed the importance of inhibition for normal sensory processing and in the development of certain pain states.

We have characterized a specific population of interneurons that express the calcium binding protein parvalbumin (PV+) (Hughes *et al*. 2012) and are broadly considered inhibitory, although a small population of excitatory PV+ INs has also been reported (Antal *et al*. 1991). Despite this, our work in the PV‐green fluorescent protein (GFP) transgenic mouse showed that GFP‐labelled PV+ INs had electrophysiological, morphological and neurochemical properties consistent with an inhibitory phenotype; and that PV‐GFP axon varicosities were vesicular GABA transporter (VGAT) positive. This work also showed that PV+ INs receive monosynaptic input from myelinated afferents, and provide a source of axo‐axonic input onto the central terminals of these afferents (Hughes *et al*. 2012). Such connectivity implies a feed‐forward inhibitory circuit could exist to selectively regulate the effect of innocuous tactile input during spinal sensory processing. It follows that a reduction in the inhibition mediated by these neurons could contribute to development of tactile allodynia. Subsequent work has verified these predictions by showing that genetic ablation of PV+ INs in the DH reduces sensory thresholds as observed in allodynia (Petitjean *et al*. 2015). Furthermore, this work showed that increased activation of PV+ INs in neuropathic mice restored normal sensory thresholds and attenuated allodynia.

Given this role for PV+ INs in modality‐specific sensory processing, the manner in which their activation or excitability is regulated will be important for developing strategies to alter the activity of this population. For example, we have shown that PV+ INs can support high frequency action potential (AP) discharge and express the hyperpolarization‐activated cation current (*I*_h_), which is associated with repetitive AP firing (Hughes *et al*. 2012). Together, these properties make PV+ INs a powerful source of inhibition in the DH. Paradoxically, we also showed that PV+ INs have a relatively high rheobase current and receive weak synaptic excitation, which imply they may be difficult to recruit.

In order to determine how PV+ INs are recruited during spinal sensory processing it is important to also fully characterize the inhibition they receive. It is well established that both GABA and glycine mediate fast synaptic inhibition in the DH (Chery & de Koninck, 1999; Graham *et al*. 2003; Baccei & Fitzgerald, 2004; Anderson *et al*. 2009; Takazawa & MacDermott, 2010) and that populations of DH neurons can receive inhibition dominated by GABA, glycine, or both. It has also been shown that a dorsoventral gradient exists in the inhibitory transmitter phenotype within the DH. GABA is more dominant in superficial layers, whereas glycine dominates in the deep DH and ventral horn (Cronin *et al*. 2004; Anderson *et al*. 2009). In addition to their role in fast synaptic inhibition, both GABA and glycine are known to mediate tonic inhibitory currents that are capable of suppressing AP discharge (Takazawa & MacDermott, 2010). Therefore, in this study we assess the levels of synaptic and tonic inhibition mediated by GABA and glycine onto PV+ INs, determine their respective roles in regulating AP discharge, and explore the stoichiometry of synaptic and extrasynaptic receptor populations. This information provides new insights on how altering GABA‐ or glycinergic transmission can affect spinal inhibition that is mediated by PV+ INs and how these mechanisms might be targeted pharmacologically.

## Methods

All experiments were approved by the University of Newcastle (UoN) Animal Care and Ethics Committee. We used a transgenic mouse line (both sexes; aged 29 ± 3 weeks; body weight 17–27 g) that expressed enhanced green fluorescent protein (eGFP) under the control of the parvalbumin promoter (PVeGFP: Meyer *et al*. 2002). The line was originally generated by Professor Hana Monyer and bred on the BalbC background with permission at UoN. UV illumination of the ears of PVeGFP mice was used to identify eGFP‐positive animals: fast twitch muscle fibres also express parvalbumin (and by association eGFP).

### Acute spinal slice preparation

Spinal cord slices were prepared using previously described techniques (Graham *et al*. 2003; Smith *et al*. 2015). Briefly, animals (PVeGFP) were anaesthetized with ketamine (100 mg kg^−1^ i.p.) and decapitated. Using a ventral approach, the lumbosacral enlargement of the spinal cord was rapidly removed and placed in ice‐cold sucrose‐substituted artificial cerebrospinal fluid (ACSF) containing (in mm): 250 sucrose, 25 NaHCO_2_, 10 glucose, 2.5 KCl, 1 NaH_2_PO_4_, 1 MgCl_2_ and 2.5 CaCl_2_. Transverse or parasagittal slices (from L3–L5 segments cut at 300 and 200 μm thickness, respectively) were obtained using a vibrating microtome (Leica VT‐1000S, Heidelberg, Germany). Slices were then transferred to an interface incubation chamber containing oxygenated ACSF (118 mm NaCl substituted for sucrose) and allowed to equilibrate for 1 h (at 22–24°C) prior to recording.

### Electrophysiology

Slices were transferred to a recording chamber and continually superperfused (bath volume 0.4 ml; exchange rate 4–6 bath volumes min^−1^) with ACSF bubbled with Carbonox (95% O_2_ and 5% CO_2_) to achieve a final pH of 7.3–7.4. Neurons were visualized using near‐infrared differential interference contrast optics. PV+ INs were identified under fluorescence using a fluorescein isothiocyanate (FITC) filter set (488 nm excitation and 508 nm emission filters) (Hughes *et al*. 2012). Neurobiotin (0.2%) was included in internal solutions for *post hoc* confirmation of GFP expression in a subset of recordings (Vector Laboratories, Peterborough, UK). As previously reported, the majority of PVeGFP‐expressing cells are immunopositive for parvalbumin (Hughes *et al*. 2012). Recordings were limited to neurons located within or close to the substantia gelatinosa. This area is easily identified by its translucent appearance in transverse and parasagittal spinal cord slices and contains a clearly discernable plexus of PV+ INs. All recordings were obtained at room temperature (21–24°C). Patch pipettes (4–8 MΩ) were filled with one of two internal solutions. A caesium chloride‐based internal was used for recording inhibitory currents. This internal contained (in mm): 130 CsCl, 10 Hepes, 10 EGTA, 1 MgCl_2_, 2 ATP and 0.3 GTP (pH adjusted to 7.35 with 1 m CsOH). A potassium gluconate‐based internal solution was used in experiments where the action potential discharge was examined and analysed. This internal contained (in mm): 135 KCH_3_SO_4_, 6 NaCl, 2 MgCl_2_, 10 Hepes, 0.1 EGTA, 2 MgATP, 0.3 NaGTP (pH adjusted to 7.3 with KOH).

All whole‐cell recordings were first established in voltage clamp (holding potential −70 mV). Data were acquired using a Multiclamp 700B amplifier (Molecular Devices, Sunnyvale, CA, USA), digitized online (sampled at 10–20 kHz and filtered at 5–10 kHz) via an ITC‐18 computer interface (Instrutech, Long Island, NY, USA) and stored on a Macintosh computer using Axograph X software (Kagi, Berkley, CA, USA). After obtaining the whole‐cell recording configuration, series resistance, neuron input resistance and membrane capacitance were calculated based on the response to a 5 mV hyperpolarizing voltage step (10 ms duration) from a holding potential of −70 mV. These values were monitored at the beginning and end of each recording session and data were rejected if values changed by more than 30%.

Miniature inhibitory postsynaptic currents (mIPSCs), which represent the postsynaptic response to spontaneous release of single vesicles of neurotransmitter (Katz & Miledi, 1969; Bekkers & Stevens, 1989; Callister & Walmsley, 1996), were pharmacologically isolated by including the sodium channel blocker tetrodotoxin (TTX; 1 μm) and the AMPA/kainate receptor antagonist 6‐cyano‐7‐nitroquinoxaline‐2,3‐dione (CNQX; 10 μm) in the bath perfusate. The currents recorded under these conditions in the mouse DH are mediated by the action of GABA, glycine, or both. In order to isolate glycinergic currents the GABA_A_ receptor antagonist bicuculline (10 μm) was next added to the bath. In a subset of experiments NMDA receptor antagonist dl‐2‐amino‐5‐phosphonovaleric acid (APV; 50 μm) was then bath applied to exclude NMDA receptor activation, but this had no effect. All remaining currents were abolished by bath application of strychnine (1 μm). At least 3 min of data were acquired under both conditions (pre‐ and post‐bicuculline application) for analysis. In some experiments picrotoxin (10 μm) and/or lindane (30 μm) was applied to test for the presence of homomeric glycine receptors. Similarly, selective glycine transporter blockers Org 25543 (10 μm) and Org 24598 (10 μm) were bath‐applied to assess the role of the neuronal and glial transporters (GlyT2 and GlyT1, respectively) in tonic current characteristics. Outside‐out membrane patches (Hamill *et al*. 1981) were excised at the conclusion of some PV+ IN recordings and exposed to bath application of 2.5–10 μm glycine to evoke single channel glycine receptor‐mediated currents. Single channel events were filtered at 1 kHz.

A separate series of experiments assessed PV+ IN AP discharge in current‐clamp mode using the potassium gluconate‐based internal solution, and bridge balance monitored throughout recordings. Neuronal excitability and AP discharge were studied by injecting a series of depolarizing step currents (800 ms duration, 20 pA increments, delivered every 8 s) into the recorded neuron when it was held at a membrane potential of −60 mV. During this protocol voltage deflections were limited, to avoid cell damage, by terminating the protocol if sustained depolarizations exceeded −20 mV (i.e. in parts of the voltage trace not containing APs). After this initial characterization of excitability glycinergic signalling was modified by either blocking glycine receptors (GlyRs) or GlyT1/GlyT2 transporters with strychnine or Org 24598 and Org 25543, respectively. Excitability was assessed when either GlyRs or the transporter were blocked.

To assess the role of endogenous glycine, electrically evoked IPSCs (eIPSCs) were elicited using a bipolar glass stimulating electrode (20 μm tip separation) positioned 200–800 μm rostral or caudal to the recorded PV+ IN. A number of stimulation protocols were applied including a single 1 ms duration stimulus, 10 stimuli at 10 Hz, 10 stimuli at 20 Hz and 20 stimuli at 20 Hz via a transistor‐transistor logic (TTL)‐driven ISO‐Flex stimulator (A.M.P.I., Jerusalem, Israel). A protocol that delivered three successive trials of depolarizing step currents (800 ms duration, 20 pA increments, delivered every 10 s) to the recorded PV+ IN was used to assess the effect of stimulation‐evoked endogenous glycine on AP discharge. Tonic glycinergic currents were evoked prior to the second depolarizing step trial (Fig. 4 *B*) using bipolar stimulation (20 stimuli, at 20 Hz). Importantly, bipolar stimulation was maintained at 10% below threshold for Na^+^ channel activation in all protocols and recordings were made in the presence of CNQX and bicuculline.

### Data analysis

Analysis of mIPSCs was completed using a sliding template method (semi‐automated procedure within Axograph software package) to detect and capture mIPSCs (Clements & Bekkers, 1997). All captured mIPSCs were inspected individually and excluded from further analysis if they contained overlapping mIPSCs or had an unstable baseline before the rise or during the decay phase of the mIPSCs. Data were also rejected if a significant trend in either mIPSC amplitude or inter‐event interval was observed during the analysis period. The peak amplitude and rise time of mIPSCs were measured for all accepted events (via semi‐automated procedures in Axograph) and instantaneous frequency was calculated as the reciprocal of inter‐event interval. Analysis of the mIPSC decay time constant (calculated over 20–80% of the decay phase) was performed on averaged mIPSCs, generated by aligning the rising phase of all accepted events in a recording. Tonic currents were analysed by calculating the change in baseline holding current and standard deviation (baseline noise) before and after bath application of bicuculline, strychnine, APV, picrotoxin, lindane, Org 24598, Org 25543, or both glycine transporter blockers. Single channel events recorded in outside‐out membrane patches were captured from continuous recordings using an amplitude threshold detection and all‐points histograms were then constructed to calculate mean single channel current and conductance for each membrane patch (*n* = 16).

In experiments that assessed the relationship between evoked endogenous glycine release and tonic glycinergic currents we measured baseline current and baseline noise over a 10 ms epoch (30–40 ms after the stimulus artefact). Measurements were made over this epoch to avoid contamination by evoked synaptic currents. Data were normalized to pre‐stimulus current values. The time for the evoked current to return to baseline was taken as the time from the stimulus artefact until the current returned to zero. In experiments involving drug application data were normalized to control responses (no drug) and traces zeroed to baseline.

AP discharge was classified according to previously published criteria (Graham *et al*. 2004, 2007). In agreement with our previous work, PV+ INs expressed either tonic discharge, characterized by persistent AP discharge throughout the depolarizing step, or initial bursting discharge, characterized by AP discharge limited to the beginning of the depolarizing step. The criterion for inclusion of a neuron in this analysis was a resting membrane potential more negative than –50 mV and a series resistance <30 MΩ (filtered at 5 kHz). In our analysis of AP discharge, individual APs elicited by step‐current injection were captured using a derivative threshold method (d*V*/d*t* ≥ 15 V s^−1^) with the inflection point during spike initiation defined as AP threshold. The difference between AP threshold and its maximum positive peak was defined as AP amplitude. AP base width was measured at AP threshold. AP after‐hyperpolarization amplitude was taken as the difference between AP threshold and the maximum negative peak following the AP. Rheobase current was defined as the smallest step current that would elicit at least one AP.

In recordings that assessed the impact of altered glycinergic inhibition on PV+ IN discharge (i.e. strychnine or glycine transporter blocker application), the impact of neuron‐to‐neuron variability in rheobase current was removed by normalization. This was achieved by normalizing responses to rheobase and then reporting subsequent responses in 20 pA increments above rheobase. In addition, the number of APs elicited and instantaneous frequency were normalized to values obtained in the 40 pA current step response under control conditions (i.e. no drug). Therefore, AP number and instantaneous frequency at this 40 pA step are reported as 100%. Drug‐induced differences in AP number and instantaneous frequency are reported as a percentage of this value. Changes in AP discharge during endogenously evoked glycine were assessed by normalizing values obtained from the second and third depolarizing step trials to data obtained from the first trial.

Single channel events recorded in outside‐out membrane patches were captured from continuous recordings using amplitude threshold detection and all‐points histograms were then constructed. To calculate mean single channel current and conductance, one or two Gaussian distributions were fitted to all‐points histograms.

All drugs were obtained from Sigma Aldrich (Sydney, Australia) unless otherwise stated.

### Statistics

Statistical analysis was carried out using SPSS v10 (SPSS Inc. Chicago, IL, USA). Student's *t* tests were used to compare variables between genotypes. Data that failed Levene's test for homogeneity of variance were compared using the non‐parametric Kruskal–Wallis test. Statistical significance was set at *P* < 0.05. All values are presented as means ± SEM.

## Results

### Glycinergic synaptic inhibition dominates in PV+ INs

**Figure 1:**
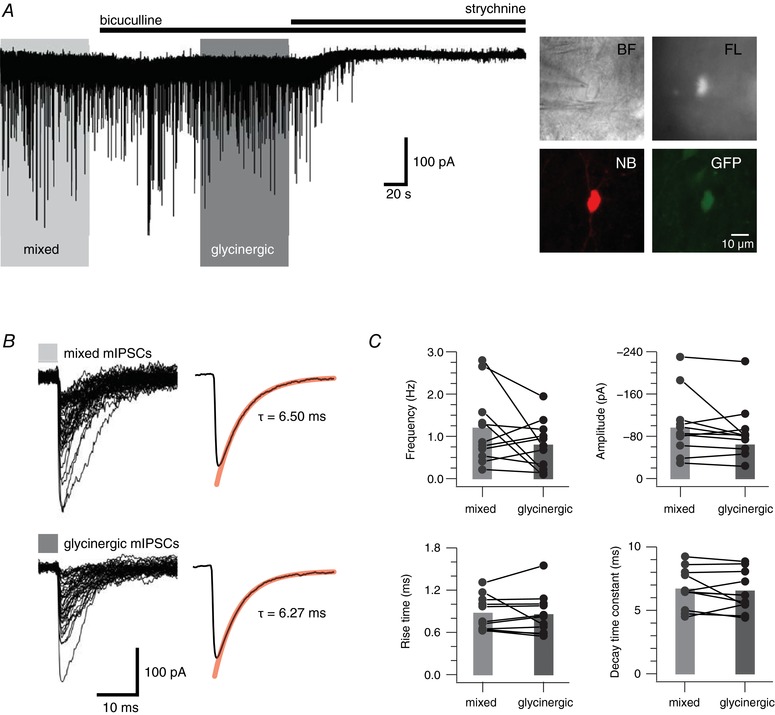
Synaptic inhibition in PV+ INs *A*, trace showing continuous mIPSC recording from a PV+ IN. Mixed mIPSCs were recorded in the presence of CNQX (10 μm) and TTX (1 μm). Glycinergic mIPSCs are revealed following bath addition of the GABA_A_ receptor antagonist bicuculline (10 μm). Bath addition of the glycine receptor antagonist strychnine (1 μm) abolished glycinergic mIPSCs. Inset, neurons were identified in infrared‐differential interference contrast (IR‐DIC) (top left), and fluorescence (top right) was subsequently used to confirm the presence of GFP. In some recordings *post hoc* recovery confirmed neurobiotin‐filled cells (bottom left) were GFP+ (bottom right). *B*, left traces show overlaid mixed and glycinergic mIPSCs captured from the recording epochs outlined by the light and dark grey rectangles in *A*. Right traces show averaged decay waveforms for mixed and glycinergic mIPSCs. Red line shows fit for decay time constant calculation. They were virtually identical for mixed and glycinergic mIPSCs. *C*, plots comparing mean mIPSC properties (frequency, amplitude, rise time and decay time constant) in PV+ INs under recording conditions that reveal mixed and glycinergic mIPSCs. The majority of recordings showed little change between conditions; however, four recordings showed a reduction in mIPSC frequency when glycinergic mIPSCs were isolated. Most PV+ INs showed little change in mIPSC amplitude, rise time and decay time constant between conditions. [Color figure can be viewed at wileyonlinelibrary.com]

To assess the relative contribution of GABA and glycine to synaptic inhibition in PV+ INs we recorded ‘mixed’ mIPSCs in the presence of CNQX and TTX (*n* = 11), and then assessed their bicuculline sensitivity (Fig. 1 *A*). Any currents remaining after the addition of bicuculline (i.e. bicuculline insensitive) were considered glycinergic. This was confirmed by bath application of the glycine receptor antagonist strychnine, which abolished all remaining activity. In most recordings from PV+ INs comparison of mixed and glycinergic mIPSCs indicated that there was little difference between these conditions (Fig. 1 *B*). Plots showing the change in mIPSC frequency, amplitude, rise time and decay time constant reveal that mixed and glycinergic mIPSC properties were relatively stable, although mIPSC frequency was reduced in 3 of 11 recordings (Fig. 1 *C*). Despite this, the mean value for frequency (1.19 ± 0.26 Hz *vs*. 0.79 ± 0.17 Hz), amplitude (109 ± 18 pA *vs*. 99 ± 17 pA), rise time (0.87 ± 0.07 ms *vs*. 0.85 ± 0.09 ms), decay time constant (6.67 ± 0.77 ms *vs*. 6.51 ± 0.48 ms) and goodness of fit of the decay time constant (assessed using the sum of squares error: 0.67 ± 0.19 *vs*. 0.64 ± 0.14) were similar in mixed and glycinergic mIPSCs. This analysis suggests glycinergic transmission is the dominant form of inhibition to PV+ INs with GABAergic synaptic transmission playing a less prominent role.

### PV+ INs receive tonic glycinergic inhibition

**Figure 2:**
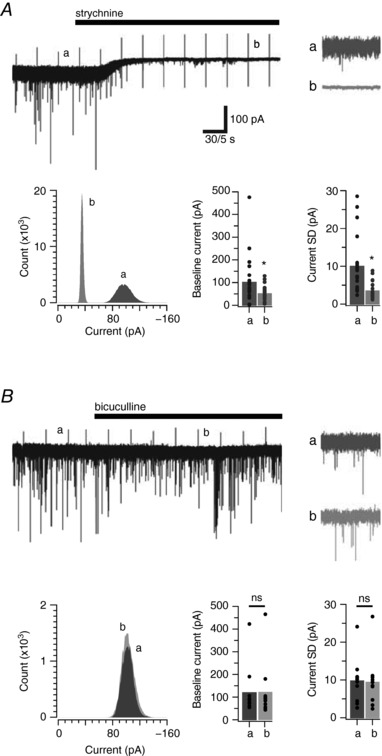
PV+ INs exhibit tonic glycine, but not GABAergic currents *A*, trace shows a continuous glycinergic mIPSC recording (in the presence of TTX, CNQX and bicuculline), before and after bath addition of strychnine. Note that in addition to abolishing glycinergic mIPSCs, strychnine causes a shift in holding current and a reduction in baseline noise. These features are indicative of a tonic glycine‐mediated current. Insets (a and b) show expanded epochs highlighting the difference in baseline noise before and after strychnine exposure. An all‐points histogram (bottom left) from the above epochs quantify the shift in holding current and reduction in baseline noise after strychnine exposure. Bar graphs (bottom right) are group data showing a significant reduction in holding current and baseline noise in PV+ INs. Data for each neuron used in mean calculation for bar graphs are shown as filled circles. *B*, same analysis as for *A*, except the continuous trace shows GABAergic mIPSCs (in the presence of TTX, CNQX and strychnine) before and after bath addition of bicuculline. Note, bicuculline abolishes all GABAergic mIPSCs but does *not* alter the holding current or baseline noise. This suggests tonic GABA currents are absent in PV+ INs. Insets (a and b) show no difference in baseline noise before or during bicuculline exposure. An all‐points histogram (bottom left) and the bar graphs (bottom right) show that holding current and baseline noise in PV+ INs do not differ before or during bicuculline exposure.

A striking feature in the mIPSC recordings made after the addition of strychnine was a clear shift in the holding current and a reduction in baseline noise (Fig. 2 *A*). This is further emphasized in all‐points histogram plots for the two epochs: before and after strychnine addition. There is a clear shift to the left (i.e. a reduction) in holding current as well as a narrowing of the distribution following the addition of strychnine. Together, these observations indicate the presence of a tonic glycinergic current under baseline conditions. Group data comparisons (*n* = 22) show that strychnine significantly reduced the holding current in our recordings by ∼50 pA (−99 ± 22 pA *vs*. −49 ± 8 pA before and after strychnine, *P* = 0.012), and the noise (standard deviation (SD)) of the baseline current (9.90 ± 1.59 pA *vs*. 3.40 ± 0.46 pA, *P* < 0.001). In contrast, there was no evidence for the existence of tonic GABAergic currents in PV+ INs after bath application of bicuculline (*n* = 11) (Fig. 2 *B*). Group comparisons of holding current (−118 ± 33 pA *vs*. −120 ± 36 pA, *P* = 0.606), and baseline current noise (9.70 ± 1.75 pA *vs*. 9.35 ± 1.96 pA, *P* = 0.46) showed no change after bicuculline application. To exclude a possible contribution of NMDA receptors to tonic currents a subset of experiments assessed the effect of APV bath application (*n* = 6; 2 mice). In no instance did APV cause a reduction in holding current (−104 ± 9 pA *vs*. −111 ± 11 pA, *P* = 0.072), or baseline current noise (6.88 ± 1.21 pA *vs*. 6.44 ± 1.04 pA, *P* = 0.254). In contrast, strychnine significantly decreased holding current (−104 ± 9 pA *vs*. −94 ± 10 pA, *P* = 0.011) and baseline current noise (6.88 ± 1.21 pA *vs*. 2.31 ± 0.27 pA, *P* = 0.009) consistent with our earlier recordings. Thus, PV+ INs in the DH express robust tonic currents mediated by glycine, but we find no evidence for the existence of tonic GABAergic or glutamatergic currents.

### Glycine transporter blockade enhances tonic currents

**Figure 3:**
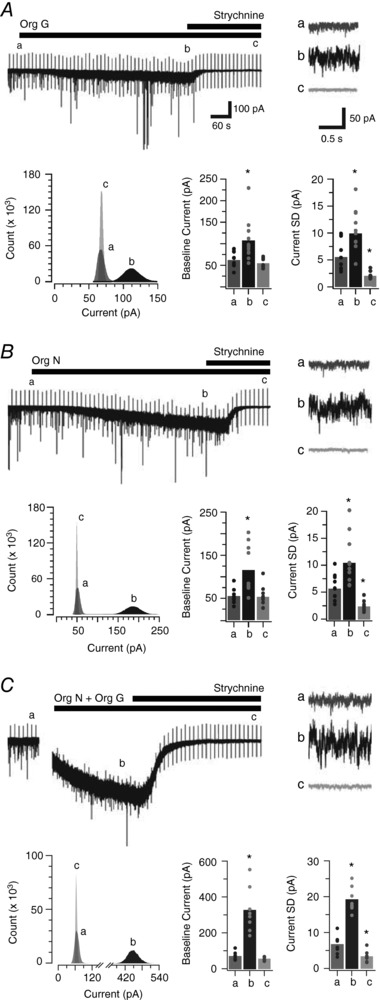
PV+ IN tonic glycine currents are enhanced by glycine transporter block *A*, trace shows a continuous glycinergic mIPSC recording (in the presence of TTX, CNQX and bicuculline) during sequential addition of the GlyT1 blocker Org 24598 (Org G) and strychnine to the bath. Org 24598 enhanced the tonic glycine current, as shown by an increased holding current and baseline noise. Addition of strychnine abolished the tonic glycinergic current. Insets (a, b and c) above the continuous mIPSC trace highlight the enhanced baseline noise after the addition of Org 24598 (b) and its reduction by strychnine (c). An all‐points histogram (bottom left) from epochs a, b and c quantifies the holding current shift, and baseline noise alterations during Org 24598 and strychnine exposure. Bar graphs (bottom right) compare group data and show that Org 24598 and strychnine shift both holding current and baseline noise in PV+ INs. *B*, trace shows a continuous recording of glycinergic mIPSCs during sequential bath application of the GlyT2 blocker Org 25543 and strychnine. Org 25543 (Org N) clearly enhanced the tonic glycine current. Insets (a, b and c) above the mIPSC trace show Org 25543 enhanced baseline noise whereas strychnine reduced it. An all‐points histogram (bottom left) from a, b and c shows the shifts in holding current and baseline noise during Org 25543 and strychnine exposure. Bar graphs (bottom right) compare group data showing Org 25543‐ and strychnine‐related holding current and baseline noise shifts in PV+ INs. *C*, trace shows a continuous glycinergic mIPSC recording, with the sequential bath addition of the GlyT1 blocker Org 24598, GlyT2 blocker Org 25543, and then strychnine. Co‐exposure to Org 24598 and Org 25543 dramatically enhanced the tonic glycine current. Insets (a, b and c) highlight the enhanced baseline noise during Org 24598 + Org 25543 application, and the strychnine‐mediated reduction. An all‐points histogram (bottom left) from these epochs quantifies the holding current shift and baseline noise alterations under Org 24598 + Org 25543 and strychnine exposure. Bar graphs (bottom right) compare group data showing Org 24598 + Org 25543‐ and strychnine‐related holding current and baseline noise shifts in PV+ INs.

To further assess the impact of altered extracellular glycine levels on tonic inhibition of PV+ INs we undertook a series of experiments that blocked the glial (GlyT1, *n* = 11; 6 mice) and neuronal (GlyT2, *n* = 10; 5 mice) glycine transporters. Bath addition of the GlyT1 blocker Org 24598 increased the holding current and baseline noise. Conversely, holding current and noise were reduced dramatically by bath application of strychnine (Fig. 3 *A*). These observations are further quantified in the all‐points histogram, which shows a rightward shift and broadening of the current distribution after addition of Org 24598. These effects were abolished after the application of strychnine. Note, the reduced amplitude of the tonic glycine currents observed here, compared to those observed in Fig. 2 *A*, emphasizes cell to cell variability in tonic glycine current amplitude and the potential for rundown associated with the longer recordings necessitated by these experiments. Bath addition of the GlyT2 blocker Org 25543 produced virtually identical results to GlyT1 blockade. It enhanced the tonic current and caused a rightward shift and broadening in the all‐points histogram (Fig. 3 *B*). These effects were completely reversed by bath application of strychnine. Group data comparisons show that block of both glial (GlyT1) and neuronal (GlyT2) transport blockers increased the holding current by approximately double (61 ± 5 pA *vs*. 106 ± 15 pA GlyT1, *P* = 0.003; 54 ± 5 pA *vs*. 116 ± 18 pA GlyT2, *P* = 0.004) as well as the associated baseline noise (SD) (5.40 ± 0.81 pA *vs*. 9.80 ± 1.10 pA GlyT1, *P* = 0.001; 5.56 ± 0.71 pA *vs*. 10.34 ± 1.39 pA GlyT2, *P* = 0.015). Thus, both glial and neuronal regulation of extrasynaptic glycine levels appear to play an important role in mediating tonic currents in PV+ INs. In a subset of experiments (*n* = 8; 4 mice) both transporters were blocked (i.e. GlyT1 and GlyT2) and, irrespective of the order of blockade, addition of the second transporter blocker caused a further increase in both holding current (67 ± 8 pA *vs*. 324 ± 44 pA, GlyT1 then GlyT2, *P* = 0.001) and baseline noise (6.57 ± 0.91 pA *vs*.19.06 ± 1.22 pA. GlyT1 then GlyT2, *P* = 0.001) (Fig. 3 *C*). This effect was greater than the summed effect of each transporter alone, suggesting that under conditions where only one transporter is blocked some residual capacity remains in the other transporter system to clear glycine from the extracellular space. Blockade of both transporters then causes a rapid elevation in glycine concentration, further enhancing tonic glycine currents and the associated holding current and baseline noise.

### Glycinergic inhibition regulates PV+ IN action potential discharge

**Figure 4:**
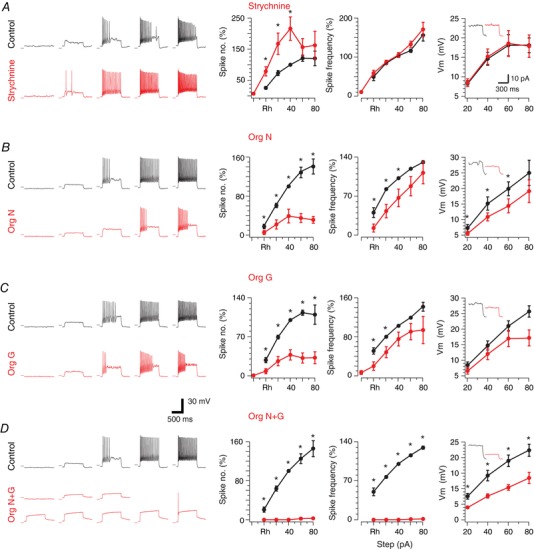
PV+ IN excitability is sensitive to levels of glycinergic inhibition *A*–*D*, left traces show action potential (AP) discharge responses of PV+ INs during depolarizing current step injections, before and after manipulation of glycinergic inhibition with strychnine or glycine transporter inhibitors. Right plots show group data summarizing AP discharge and frequency per step above rheobase current, as well as subthreshold current–voltage (*I–V*) relationships. *A*, strychnine antagonism of glycine receptors increased AP discharge. This increase is evident in the relationship between AP number *versus* current step (left); however, AP discharge frequency (middle) was not changed by strychnine. *B*, traces show AP discharge decreased after GlyT1 blocker Org 24598 exposure. This decrease is clear in the group data plots of AP discharge and frequency per step above rheobase. *C*, traces show AP discharge decreased after GlyT2 blocker Org 25543 exposure. This decrease is clear in group data plots of AP discharge and frequency per step above rheobase. *D*, traces show combined GlyT1 and GlyT2 block with Org 24598 and Org 25543 dramatically decreased AP discharge. This dramatic effect is clear in group data plots of AP discharge and frequency per step above rheobase. Insets in *I–V* plots show example responses to 40 pA depolarizing current steps under control conditions and in the presence of glycine receptor antagonist or inhibitors. [Color figure can be viewed at wileyonlinelibrary.com]

In order to test the functional consequences of the dominant glycinergic inhibitory control of PV+ INs, we assessed their intrinsic excitability before and after manipulation of glycinergic input. PV+ IN discharge was first assessed by recording the AP discharge evoked during injection of a series of depolarizing current steps. Unlike classical fast spiking discharge described for PV+ INs in higher CNS regions (hippocampus, cortex) spinal DH PV + IN discharge could be classified as either tonic firing, characterized by sustained discharge for the duration of current injection; or initial bursting, where discharge was limited to the beginning of current injection; consistent with our previous report (Hughes *et al*. 2012). Under control conditions, both the number of evoked APs and mean instantaneous firing frequency (*F*) increased with increasing levels of current injection (from 0 to 100 pA). These data are combined and presented as *F*–*I* plots in Fig. 4 with values normalized to the level of discharge evoked by the 40 pA current step (above rheobase) under control conditions. After this initial trial we either blocked glycinergic inhibition by bath‐applying strychnine (1 μm), or enhanced it by applying the GlyT1 blocker and/or GlyT2 blocker. Action potential discharge was then reassessed 3 min after drug application from the same control membrane potential.

In the strychnine experiments (*n* = 14; 6 mice) half the recordings initially exhibited tonic firing and half showed initial bursting discharge. Comparisons of control and strychnine data showed a significant shift to the left in the *F–I* curve indicating PV+ INs became more excitable (Fig. 4 *A*). AP discharge rate more than doubled (216 ± 38%, when normalized to the 40 pA step, *P* = 0.009). In contrast, mean instantaneous frequency did not change (102 ± 6%, normalized to the 40 pA step, *P* = 0.754). In some initial bursting neurons (5/7) addition of strychnine changed or ‘converted’ PV+ IN discharge to the tonic firing mode. Likewise, strychnine altered the discharge of tonic firing neurons by increasing the number of APs discharged per step, without converting firing mode. These data suggest that under our recording conditions initial bursting neurons are strongly influenced by glycinergic inhibition, and its removal increases their capacity to support sustained AP discharge. To further explore the lack or small effect of glycinergic inhibition on tonic firing neurons we increased extracellular glycine concentration by bath‐applying Org 24598 (*n* = 11; 3 mice) and Org 25543 (*n* = 11; 8 mice) either independently or together (*n* = 9; 5 mice). In these GlyT blocker experiments, this manipulation caused a rightward shift in the *F–I* plots for *both* tonic firing (*n* = 16) and initial bursting (*n* = 7) neurons (Fig. 4 *B*–*D*). Group data comparisons show that application of glycine transport blockers decreased the number of action potentials below baseline values (38 ± 9% GlyT1, *P* = 0.000; 39 ± 14% GlyT2, *P* = 0.002; 0 ± 0% GlyT1 + GlyT2, *P* = 0.001, normalized to values at 40 pA current step) and also the instantaneous frequency (73 ± 14% GlyT1, *P* = 0.086; 65 ± 16% GlyT2, *P* = 0.049; 0 ± 0% GlyT1 + GlyT2, *P* = 0.001). In most cases the tonic firing discharge pattern was converted to initial bursting or single spiking (17/21) and the initial bursting discharge pattern converted to single spiking or reluctant firing (6/10) following GlyT block. Thus, glycine levels and the resulting inhibition play an important role in shaping ‘firing mode’ in *both* tonic firing and initial bursting PV+ INs.

In an attempt to explain the observed changes in spike number and frequency when glycine levels are manipulated we examined the properties of the APs recorded at spike threshold. Comparisons of the pre‐ and post‐strychnine data showed rheobase current was unchanged (105 ± 14%, *P* = 0.726), whereas application of GlyT blockers increased rheobase current (136 ± 12% for GlyT1, *P* = 0.012; 178 ± 32% GlyT2, *P* = 0.039; 328 ± 71% GlyT1 + GlyT2, *P* = 0.012). Strychnine application caused an increase in AP height (119 ± 5%, *P* = 0.003), but no change in AP half‐width (104 ± 3%, *P* = 0.141), or after‐hyperpolarization peak (102 ± 9%, *P* = 0.835). For the most part, application of GlyT blockers resulted in no change to AP peak amplitude (89 ± 3% GlyT1, *P* = 0.010; 104 ± 4% GlyT2, *P* = 0.334; 97 ± 2% GlyT1 + GlyT2, *P* = 0.191), a slight increase in AP half‐width (115 ± 9% GlyT1, *P* = 0.136; 105 ± 4% GlyT2, *P* = 0.281; 112 ± 3% GlyT1 + GlyT2, *P* = 0.004), and little change in after‐hyperpolarization peak amplitude (96 ± 10% GlyT1, *P* = 0.662; 86 ± 8% GlyT2, *P* = 0.130; 76 ± 11% GlyT1 + GlyT2, *P* = 0.069). The clear increase in rheobase current helps explain the observed changes in AP number and frequency. Simply put, enhanced glycine receptor activation reduces neuronal input resistance (74 ± 6% GlyT1, *P* = 0.002; 84 ± 9% GlyT2, *P* = 0.09; 71 ± 9% GlyT1 + GlyT2, *P* = 0.13) and necessitates increased levels of current injection to reach spike threshold. To further examine this we analysed the subthreshold voltage deflections during depolarizing current injection steps (Fig. 4). Application of strychnine (*n* = 12) had no effect on subthreshold voltage responses, whereas GlyT block reduced subthreshold voltage responses (68 ± 11% GlyT1, *P* = 0.046; 76 ± 8% GlyT2, *P* = 0.06; 62 ± 8% GlyT1 + GlyT2, *P* = 0.015 at 80 pA injection) and caused a right‐shift in current–voltage (*I–V*) relationships.

### Synaptically evoked tonic glycine currents

**Figure 5:**
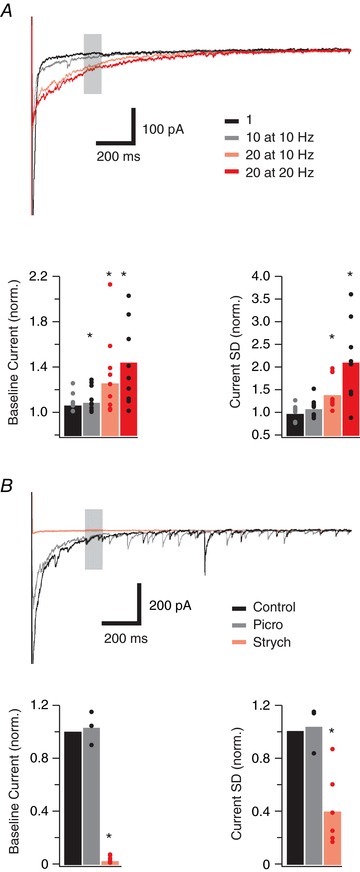
Evoked (endogenous) glycine release enhances tonic glycine currents *A*, top current traces show the recovery phase recorded following a series of glycinergic eIPSCs evoked by stimulation (bipolar electrode) of glycinergic afferents at the lamina II/III boundary, in the presence of CNQX and bicuculline. Four stimulus protocols were employed to produce increasing glycine release: 1 stimulus (black trace), 10 stimuli at 10 Hz (grey trace), 10 stimuli at 20 Hz (pink trace), 20 stimuli at 20 Hz (red trace). Data were obtained from a 10 ms epoch 30–40 ms after the stimulus artifact (shaded area). Note that increasing levels of stimulation cause an increase in the amplitude and duration of the evoked current. Bar graphs (bottom) show changes in three current properties (baseline current and current SD) as stimulus intensity is increased. *B*, top current traces show the recovery phase after glycinergic eIPSCs (20 stimuli at 20 Hz) under control conditions (black trace), after addition of picrotoxin (grey trace), and after addition of strychnine (red trace). Note, picrotoxin did not affect the response whereas strychnine abolished response to electrical stimulation. Bar graphs (below) show group data confirming picrotoxin (grey) has no effect on the eIPSC‐related tonic current, whereas addition of strychnine (pink) abolishes the response. [Color figure can be viewed at wileyonlinelibrary.com]

To examine whether endogenously released glycine contributed to tonic currents in PV+ INs we electrically evoked glycinergic IPSCs using bipolar stimulation in the presence of CNQX and bicuculline (*n* = 10; 2 mice). Electrical stimulation resulted in a long lasting enhancement of the tonic current (sometimes >1 s) seen as increased holding current and baseline noise that was sensitive to stimulus duration (Fig. 5 *A*). From 30 to 40 ms post‐stimulation; a single eIPSC did not significantly increase holding current (191 ± 67 pA *vs*. 208 ± 72 pA, *P* = 0.059), baseline noise (9.59 ± 3.21 pA *vs*. 10.32 ± 2.38 pA, *P* = 0.628), and returned to baseline in 0.69 ± 0.14 s; 10 eIPSCs at 10 Hz increased holding current (141 ± 22 pA *vs*. 153 ± 23 pA, *P* = 0.021), but not baseline noise (6.14 ± 1.29 pA *vs*. 6.32 ± 1.21 pA, *P* = 0.291), and returned to baseline in 0.85 ± 0.19 s; 10 eIPSCs at 20 Hz increased holding current (224 ± 86 pA *vs*. 281 ± 114 pA, *P* = 0.032), baseline noise (8.26 ± 2.01 pA *vs*. 12.12 ± 3.68 pA, *P* = 0.009), and returned to baseline in 1.14 ± 0.25 s; 20 eIPSCs at 20 Hz increased holding current (174 ± 37 pA *vs*. 255 ± 79 pA, *P* = 0.011), baseline noise (7.29 ± 1.71 pA *vs*. 16.65 ± 5.91 pA, *P* = 0.005), and returned to baseline in 1.23 ± 0.23 s. Bath addition of picrotoxin (Fig. 5 *B*; *n* = 3; 1 mouse) did not affect the stimulus‐evoked changes to holding current (97 ± 30 pA *vs*. 91 ± 28 pA, *P* = 0.713), baseline noise (11.72 ± 3.12 pA *vs*. 12.52 ± 4.06 pA, *P* = 0.619), or time to baseline (0.96 ± 0.28 s *vs*. 0.90 ± 0.25 s, *P* = 0.433). In contrast, these effects were completely abolished by bath application of strychnine (*n* = 6; 2 mice) holding current (97 ± 30 pA *vs*. 4 ± 2 pA, *P* < 0.001), baseline noise (11.23 ± 3.03 pA *vs*. 2.82 ± 0.17 pA, *P* = 0.001), and reduced time to baseline (1.05 ± 0.30 s *vs*. 0.11 ± 0.05 s, *P* < 0.001). Thus, synaptic glycine coming from eIPSCs enhanced the tonic glycinergic current.

**Figure 6:**
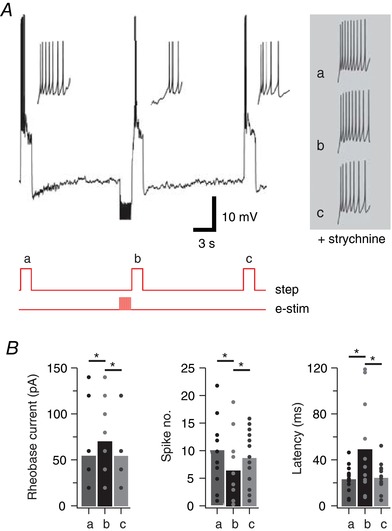
Evoked (endogenous) glycine release alters AP discharge in PV+ INs *A*, top trace shows AP discharge responses to three depolarizing step injections: (a) pre‐test; step; (b) test step – preceded by a barrage of eIPSCs (20 stimuli at 20 Hz via bipolar electrode); and (c) post‐test step. Traces below show current step injection and electrical stimulation timing (red). Insets show the onset of AP discharge on expanded time scale highlighting the delay to AP discharge and reduced AP number in the test response (b). Right traces shows expanded responses to the same protocol repeated in the presence of strychnine (1 μm), which blocks glycinergic eIPSCs and associated tonic current. Strychnine abolishes the delayed and reduced AP discharge in test step. *B*, bar graphs show group data comparing rheobase current, AP number and AP latency between the pre‐test, test and post‐test step responses, confirming that eIPSCs and the resulting tonic current reduced the excitability by modifying AP discharge properties in PV+ INs. [Color figure can be viewed at wileyonlinelibrary.com]

To assess the functional consequences of synaptically enhanced tonic glycine currents on PV+ IN AP discharge, we examined excitability with (test) and without (pre‐test and post‐test) a preconditioning eIPSC input (Fig. 6; *n* = 13; 4 mice). In these experiments, AP discharge was altered by the preconditioning eIPSCs in 13/34 neurons, supporting a tonic glycine current effect. Comparisons between depolarizing step current without (pre‐test) and with (test) preceding eIPSCs (Fig. 6 *B*) showed eIPSCs increased rheobase current (55 ± 10 pA for pre‐test *vs*. 71 ± 11 pA for test, *P* = 0.035 *vs*. 55 ± 9 pA for post‐test, *P* = 0.035), with an associated increase in AP threshold (−34.15 ± 1.83 pA for pre‐test *vs*. −31.79 ± 1.97 pA for test, *P* = 0.002 *vs*. −33.46 ± 1.89 pA for post‐test, *P* = 0.032, not shown). We also assessed changes to AP number, frequency and latency at rheobase + 20 pA (pre‐test). Group comparisons show that preceding eIPSCs reduced action potential number by 38 ± 18% (10.3 ± 1.9, pre‐test *vs*. 6.6 ± 1.8, test, *P* = 0.003 *vs*. 8.9 ± 1.4, post‐test, *P* = 0.005) and increased latency to first AP by 193 ± 33% (24.3 ± 3.2 ms, pre‐test *vs*. 50.2 ± 12.4 ms, test, *P* = 0.018 *vs*. 25.6 ± 3.4 ms, post‐test, *P* = 0.043). In contrast, preceding eIPSCs did not change mean AP frequency (34.6 ± 1.3 Hz, pre‐test *vs*. 30.6 ± 2.6 Hz, test, *P* = 0.075 *vs*. 32.9 ± 1.5 Hz, post‐test, *P* = 0.222, not shown). Importantly, strychnine (*n* = 4; 1 mouse) abolished the preconditioning eIPSC effect; rheobase (55 ± 9.6 pA, pre‐test *vs*. 60 ± 11.5 pA, test, *P* = 0.391 *vs*. 65 ± 15 pA, post‐test, *P* = 0.391); spike threshold (−32.08 ± 3.52 pA, pre‐test *vs*. −31.38 ± 3.24 pA, test, *P* = 0.095 *vs*. −31.97 ± 3.51 pA, post‐test, *P* = 0.321); action potential number (19.3 ± 7.7, pre‐test *vs*. 14.3 ± 5.4, test, *P* = 0.906 *vs*. 12 ± 6.1, post‐test, *P* = 0.310); mean frequency (40 ± 4.3 Hz, pre‐test *vs*. 37.3 ± 4 Hz, test, *P* = 0.077 *vs*. 31.3 ± 4.7 Hz, post‐test, *P* = 0.192); latency to first spike (21.6 ± 5.5 ms, pre‐test; 21.5 ± 5.9 ms, test, *P* = 0.742 *vs*. 25.7 ± 6.7 ms, post‐test, *P* = 0.068). Thus, endogenously released glycine is capable of modulating PV+ IN excitability.

### Subunit composition of tonic and synaptic glycine receptors

It is well established that differences in GABA_A_ receptor subunit composition determine whether receptors are localized to synaptic or extrasynaptic sites, and therefore contribute to phasic or tonic inhibition (Brickley & Mody, 2012). The same information is not available for glycine receptors. Given PV+ INs are under strong synaptic and tonic glycinergic inhibition, they represent an ideal model to test the relationship between glycine receptor composition and synaptic/extrasynaptic localization. For glycine receptors, it is well established that incorporation of the β subunit in heteromeric glycine receptors is critical for synaptic stabilization via the β subunit's interactions with the cytoskeletal binding protein gephyrin (Geiman *et al*. 2002). Conversely, homomeric glycine receptors, composed of five α subunits, do not interact with gephyrin and are therefore more likely to be localized extrasynaptically.

**Figure 7:**
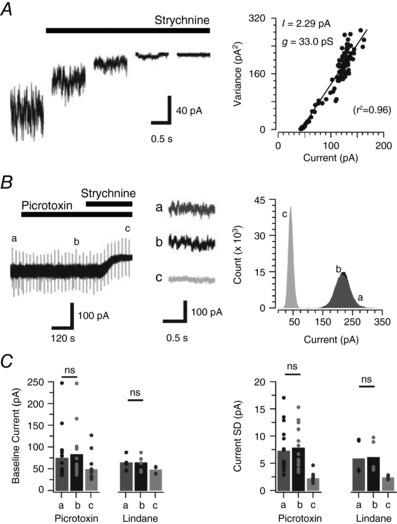
Heteromeric glycine receptors mediate tonic glycine currents in PV+ INs *A*, traces (1 s epochs) extracted from progressive block of a tonic glycine current via strychnine application (1 μm). Non‐stationary noise analysis on the current/variance relationship during the progressive strychnine block (right) was used to estimate the mean single channel conductance of underlying receptors. *B*, trace shows a continuous recording of mIPSCs (in the presence of TTX, CNQX and bicuculline), during sequential bath addition of picrotoxin and strychnine (1 μm). Note, picrotoxin does not affect the holding current or baseline noise in PV+ INs. In contrast, strychnine causes a shift in holding current and reduces baseline noise, thus confirming the presence of a tonic glycine current. Right insets (a, b and c) show no change in baseline noise after the addition of picrotoxin (b) and its reduction by strychnine (c). An all‐points histogram (right) from epochs a, b and c shows that holding current and baseline noise do not differ during picrotoxin exposure, but are reduced following addition of strychnine. *C*, bar plots showing group data compare holding current amplitude and baseline noise (current SD) under control conditions (a), following bath application of picrotoxin or lindane (b), and finally following strychnine application (c). Picrotoxin and lindane did not alter tonic current properties suggesting these currents are mediated by heteromeric GlyRs in PV+ INs.

We set out to test if the glycine receptors mediating tonic currents in PV+ INs had heteromeric α/β, or homomeric α only subunit composition. Importantly, heteromeric and homomeric glycine receptors can be differentiated by their mean single channel conductance (heteromeric < homomeric). Non‐stationary fluctuation analysis of tonic glycinergic currents during bath‐applied strychnine (Fig. 7 *A*) estimated a relatively low mean single channel conductance of 28.8 ± 0.9 pS (*n* = 19), which is consistent with heteromeric glycine receptors. This finding was reinforced by the observation that picrotoxin and lindane, which show selectivity for homomeric glycine receptors, had no effect on tonic glycinergic current amplitude (picrotoxin: −73.5 ± 14.1 *vs*. 81.8 ± 14.0 pA, *n* = 16, *P* = 0.1; lindane: −62.9 ± 7.7 *vs*. −64.5 ± 8.2 pA, *n* = 5, *P* = 0.4) or noise level (picrotoxin: 7.2 ± 1.1 *vs*. 7.7 ± 1.0 pA, *n* = 16, *P* = 0.101; lindane: 6.1 ± 1.4 *vs*. 6.3 ± 1.3 pA, *n* = 5, *P* = 0.2) (Fig. 7 *B* and *C*). These findings suggest that the tonic glycinergic currents observed in PV+ INs are not mediated by high‐conductance homomeric glycine receptors. Rather, they are composed of heteromeric glycine receptors.

**Figure 8:**
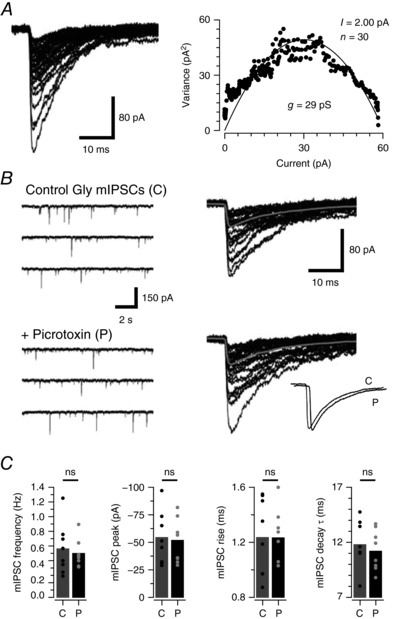
Heteromeric glycine receptors mediate synaptic glycine currents in PV+ INs *A*, glycinergic mIPSCs captured from PV+ INs (overlaid traces) were used to undertake peak‐scaled non‐stationary noise analysis and subsequently estimate mean single channel conductance of synaptically located glycine receptors (right plot). PV+ INs exhibited relatively low conductances, which is consistent with the presence of heteromeric α/β glycine receptors. *B*, continuous glycinergic mIPSC recordings prior to (control) and during picrotoxin application (left) and overlaid mIPSCs captured under each condition. Inset (bottom right) compares averaged mIPSCs recorded in control and picrotoxin (traces offset for comparison). *C*, bar plots compare group data for mIPSC frequency, amplitude, rise time and decay time constant under control conditions (C) and in picrotoxin (P). Values for individual neurons are shown as filled circles. Picrotoxin did not alter mean mIPSC properties, thus confirming these currents are mediated by heteromeric α/β GlyRs in PV+ INs.

**Figure 9:**
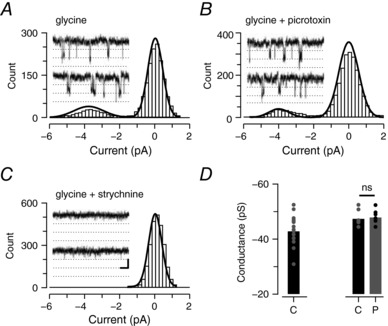
Heteromeric glycine receptors mediate glycinergic currents in PV+ INs *A*–*C*, plots showing all‐points histograms generated from single channel recordings in outside‐out membrane patches from PV+ INs during bath application of glycine. Insets are example traces from the same patch, dotted line is 2 pA. *A*, plot shows single channel events after addition of glycine. *B*, plot shows that addition of picrotoxin (10 μm) has no effect on channel conductance. *C*, plot shows addition of strychnine abolishes single channel events. *D*, bar plots showing single channel conductance from all recorded patches (left bar) and the effect of picrotoxin on single channel conductance (right, control (C) *vs*. picrotoxin (P)) in a subset of recordings. The relatively low mean single channel conductance and picrotoxin resistance obtained from this analysis supports the presence of heteromeric α/β GlyRs in the patches.

In order to confirm the synaptic glycine receptors were also heteromeric, we under took additional experiments to determine the mean single channel conductance of synaptically located glycine receptors and test the picrotoxin sensitivity of glycinergic mIPSCs. Peak scaled non‐stationary noise analysis on the decay phase of glycinergic mIPSCs estimated a relatively low mean single channel conductance of 28.84 ± 0.78 pS (*n* = 19). This supports heteromeric α/β subunit composition at synapses (Fig. 8 *A*). Likewise, picrotoxin had no effect on mIPSC frequency (0.60 ± 0.1 *vs*. 0.54 ± 0.1 Hz, *n* = 8, *P* = 0.39), amplitude (−54.2 ± 8.3 *vs*. −51.6 ± 7.0 pA, *n* = 8, *P* = 0.51), rise time (1.2 ± 0.1 *vs*. 1.2 ± 0.1 ms, *n* = 8, *P* = 0.89), or decay time constant (11.7 ± 0.8 *vs*. 11.2 ± 0.7 ms, *n* = 8, *P* = 0.22) (Fig. 8 *B* and *C*). Thus, we conclude that synaptic glycine receptors in PV+ INs are also composed of α/β subunit‐containing heteromers. Finally, the single channel conductance of glycine receptors was directly assessed in outside‐out membrane patches from PV+ INs during bath‐applied glycine (2.5–10 μm) (Fig. 9). Under these conditions single channel events evoked by glycine application had a mean conductance of 42.83 ± 1.38 pS (*n* = 16), were picrotoxin insensitive (42.83 ± 1.38 pS *vs*. 47.94 ± 1.15 pS, *n* = 6) and blocked by strychnine (*n* = 6). These data are consistent with the main conductance state of previously recorded single channel currents within the spinal cord (Bormann *et al*. 1987; Takahashi *et al*. 1992) and provide direct evidence of heteromeric glycine receptor composition in PV+ INs (Lynch, 2009).

## Discussion

This study has targeted PV+ INs in the mouse DH and shows that multiple forms of glycinergic inhibition regulate the activity of this important population. We show that synaptic inhibition of PV+ INs is dominated by glycinergic sources, and that tonic glycinergic currents are also strongly expressed in this population. Given this configuration, we assessed the stoichiometry of glycine receptors underlying synaptic and tonic currents and show that, surprisingly, heteromeric α/β subunit‐containing receptors dominate in both locations. Functionally, our data emphasize the importance of glycinergic inhibition for shaping AP discharge in PV+ INs, because enhancing or diminishing this inhibition can decrease or increase PV+ IN excitability, respectively. Given the importance of this population in setting mechanical thresholds and allodynia (Petitjean *et al*. 2015) these new data provide insights on strategies for selectively targeting the PV+ IN population pharmacologically through glycine receptors.

Inhibition in the DH has long been of interest as it is well accepted that enhanced inhibition in this region can reduce nociceptive signalling and produce analgesia (Zeilhofer *et al*. 2012). Throughout the spinal cord both GABA and glycine mediate fast synaptic inhibition and an extensive literature describes DH neurons under GABAergic, glycinergic, or mixed (GABAergic and glycinergic) inhibitory control. In general, GABAergic inhibition is more prominent in superficial laminae, while glycinergic inhibition dominates in deeper laminae (Cronin *et al*. 2004; Anderson *et al*. 2009; Zeilhofer *et al*. 2012). This mirrors the distribution of glycinergic and GABAergic interneurons within the DH, with GABAergic populations being widespread in superficial laminae and glycinergic neurons concentrated in the deep DH (Todd, 2010; Polgar *et al*. 2013). On a more detailed level, some work has reported that glycinergic inhibition dominates in lamina I of the rat, with GABAergic inhibition being more prominent in lamina II (Chery & de Koninck, 1999). Our work in the mouse, albeit not directly distinguishing lamina I and lamina II, has not identified a preferential role for glycinergic input to the most superficial regions, nor exclusively GABAergic input in substantia gelatinosa (Graham *et al*. 2003, 2011). The PV+ INs studied in the current experiments are located in laminae IIi and III and therefore lie at the border of superficial and deep DH (Hughes *et al*. 2012). Thus, the plexus of PV+ INs in this region may represent a more widespread transition to glycinergic dominance within the DH. Future studies assessing inhibition to PV‐negative DH neurons in the same region will be required to test this demarcation. Regardless, the strong glycinergic input to PV+ INs has functional implications, as the temporal properties of glycinergic and GABAergic currents in this region differ. Synaptic currents mediated by glycine have fast kinetics with a particularly rapid decay time course (Lynch, 2004). This feature, at least in the ventral horn, is thought to suit strong and precisely timed inhibition in neural circuits associated with locomotor pattern generation (Callister & Graham, 2010; Fink *et al*. 2014). Previous work from our group and others has identified a number of postsynaptic targets for the PV‐expressing population: (i) axo‐axonic synapses onto the central terminals of myelinated afferents that relay innocuous tactile input to the DH (Hughes *et al*. 2012); (ii) axo‐dendritic synapses to mediate the postsynaptic inhibition of other PV‐immunoreactive interneurons (Hughes *et al*. 2012); and (iii) PKCγ‐expressing excitatory interneurons, that are critical for the relay of innocuous tactile input from the deep to superficial DH (Petitjean *et al*. 2015). The relative expression pattern of axon terminals enriched in GABA and glycine, their respective receptor subunits, or their associated anchoring proteins within the spinal cord also imply functional differences between the inhibition mediated by GABA and glycine, respectively. GABA‐mediated inhibition appears to predominate in presynaptic (axo‐axonic) inhibition, and glycinergic inhibition is more important in postsynaptic (axo‐dendritic or axo‐somatic) inhibition (Todd, 1996; Watson & Bazzaz, 2001; Geiman *et al*. 2002; Watson *et al*. 2002; Watson, 2003; Lorenzo *et al*. 2014), Therefore, both the axo‐axonic and axo‐dendritic inhibitory inputs mediated by PV+ INs appear to have distinct roles in gating somatosensory input to ensure that innocuous tactile afferents do not excite nociceptive circuits. The temporal precision of glycinergic inhibition to control PV+ IN activity may therefore be advantageous for this regulation.

Interest in the role of glycinergic inhibition in pain circuits has increased since reports that receptors containing the α3 subunit of the glycine receptor are selectively expressed in lamina II (Harvey *et al*. 2004), where PV+ INs and their axonal arbors are concentrated (Hughes *et al*. 2012). These α3 subunit‐containing receptors can be phosphorylated by PKA‐dependent prostaglandin E2 signalling, which results in reduced glycinergic inhibition and inflammatory pain. It remains to be determined whether α3 subunit‐containing receptors are involved in glycinergic inhibition on PV+ INs; however, knockout of the α3 subunit selectively abolishes inflammatory pain. If PV+ INs do express substantial levels of α3‐containing glycine receptors this may contribute to the observed reduction of inflammatory pain because α3 knockout would enhance PV+ IN‐mediated inhibition. Future studies assessing α3 glycine receptor subunit expression at an ultrastructural or molecular level will be required to resolve the relevance of these observations for PV+ INs.

In addition to inhibitory synaptic input, a striking feature of our recordings was the presence of robust tonic glycinergic currents in PV+ INs (Fig. 2 *A*). Among recordings there was variability in the size of these currents, indicating variation within the PV+ IN population. Whether this variation is due to receptor expression and density or localized extracellular glycine concentration remains to be determined. In contrast, we found no evidence of tonic GABA currents in PV+ INs. Tonic GABAergic currents are well described in many regions of the CNS and are thought to reflect the stochastic activation of GABA_A_ receptors by basal levels of extracellular GABA (Farrant & Nusser, 2005). Consensus in the field indicates that these tonic currents are predominantly mediated by high affinity GABA_A_ receptors containing δ or α5 subunits (Brickley & Mody, 2012). This literature underpins the view that GABA_A_ receptors are selectively trafficked to either synaptic or extrasynaptic locations based on their subunit composition. GABAergic, as well as glycinergic, tonic currents have also been described within the spinal dorsal horn (Takahashi *et al*. 2006; Takazawa & MacDermott, 2010). For tonic GABAergic currents, it has been confirmed that both δ and α5 subunit‐containing receptors are crucial (Bonin *et al*. 2011; Bravo‐Hernández *et al*. 2016; Perez‐Sanchez *et al*. 2017), tonic rather than synaptic inhibition is responsible for the major effect of neurosteroids on GABA_A_ receptors (Mitchell *et al*. 2007), and that each receptor type is functionally relevant for nociceptive processing. This work suggests tonic GABAergic currents play roles in normal resolution of hyperalgesia following acute inflammation (Perez‐Sanchez *et al*. 2017), as well as limiting acute nociception and central sensitization (Bonin *et al*. 2011). In contrast, some work suggests that tonic GABAergic currents can contribute to more chronic forms of pain following inflammation and nerve injury (Bravo‐Hernández *et al*. 2016). Less work has assessed the properties and roles of tonic glycinergic currents in the dorsal horn and thus the potential for different receptor stoichiometry between extrasynaptic *versus* synaptic GlyR pools has remained unresolved. Despite this, it is widely accepted that synaptic stabilization of GlyRs requires interactions between the β‐subunit and the scaffolding protein gephyrin, implying synaptic inhibition is mediated by heteromeric α/β GlyRs (Graham *et al*. 2006; Lynch, 2009). By extension, it has been assumed that homomeric GlyRα receptors preferentially contribute to tonic inhibition. In contrast our data indicate that, at least in the PV+ IN population, heteromeric α/β GlyRs mediate both synaptic and tonic inhibition. Therefore, the relative expression of GlyRs at synaptic *versus* extrasynaptic sites appears to be a function of synaptic stabilization sites (i.e. gephyrin expression) and the overall expression of heteromeric α/β GlyRs. Thus, regulation of the subcellular distribution of GlyRs differs from GABA_A_Rs and is not dictated by subunit stoichiometry. Future work in other DH populations will be required to determine if this is a fundamental difference between receptor types, or specific to the PV+ IN population.

Another study in the glutamate decarboxylase 67 (GAD67)‐GFP mouse established more broadly that inhibitory interneurons in the DH can express either GABAergic or glycinergic tonic currents (Takazawa & MacDermott, 2010). This work showed that inhibitory neurons at the lamina II/III border were more likely to express tonic glycinergic than GABAergic currents, and that the glial glycine transporter (GlyT1) played a role in regulating tonic glycinergic currents. The similarity of these observations to our own raises the possibility that PV+ INs may have been included in the GAD67‐GFP dataset. Our experiments have extended these findings showing that both GlyT1 and the neuronal glycine transporter (GlyT2) have powerful effects on tonic glycinergic currents in PV+ INs. The role of each transporter appears similar, as we found the change in tonic current amplitude and baseline noise to be similar regardless of which transporter was blocked. Our sequential blocking experiments demonstrate a dramatic effect on tonic currents when both transporters are blocked (Fig. 3), greater than the simple sum of each transporter's effect. Furthermore, this effect was not altered by the order of blockade (i.e. GlyT1 block followed by GlyT2 block, or GlyT2 block followed by GlyT1 block). These findings imply that when a single transporter is blocked, the alternative transporter can partially compensate, irrespective of order. Once both transporters are blocked, however, a rapid rise in extracellular glycine concentration can powerfully augment the tonic glycinergic current.

Regarding the functional relevance of tonic glycine currents, tonic GABA currents in the cerebellum, hippocampus, olfactory bulb and cortex have been shown to regulate neuronal excitability by modulating neuronal input resistance and membrane time constant to alter action potential threshold, discharge pattern and input/output gain (Semyanov *et al*. 2004; Brickley & Mody, 2012). Studies on tonic currents in DH neurons recorded from GAD67‐GFP mice have also shown that blocking both GABA and glycine receptors alters neuronal excitability to enhance action potential discharge (Takazawa & MacDermott, 2010). However, these recordings were made from a heterogeneous population of inhibitory interneurons and did not differentiate between the effect of tonic GABA and glycine currents. Our targeted experiments in PV+ INs show that glycine receptor block enhances neuronal excitability in this population. This was most striking when the initial bursting discharge mode was converted to tonic firing after glycine receptor block. It is unclear from our data whether this effect was mediated by block of synaptic or tonic currents, as there is no way to selectively block each form of inhibition. The sustained *versus* phasic nature of tonic and synaptic inhibition, argues that tonic glycine currents play a critical role in this phenomenon (Farrant & Nusser, 2005).

Experiments that enhanced glycinergic inhibition by blocking glycine transporter activity reduced PV+ IN excitability. This is predictable as increasing tonic glycine currents should lower neuronal input resistance, as has been shown for tonic GABA currents, and increase the level of depolarization required to reach action potential threshold. In many instances (17/21) transporter blockade converted tonic firing into initial bursting, and when both transporters were blocked AP discharge was almost completely abolished. These findings are particularly relevant in light of the recent interest in glycine transporter blockers as potential analgesics (Vandenberg *et al*. 2014). A number of preclinical studies have confirmed that intravenous or intrathecal administration of either GlyT1 or GlyT2 blockers provides analgesia in models of acute and neuropathic pain (Morita *et al*. 2008; Tanabe *et al*. 2008; Haranishi *et al*. 2010; Nishikawa *et al*. 2010). The GlyT2 blockers are considered more promising targets because their expression is limited to inhibitory interneurons and their effects are confined to inhibitory synapses. In contrast, GlyT1 expression is largely glial and affects inhibitory (glycinergic) synapses, as well as excitatory synapses through NMDA receptor activation (Vandenberg *et al*. 2014). The premise behind these findings is that glycine transporter block increases extracellular glycine concentrations to enhance glycine receptor activation and signalling, and thus reduce nociceptive transmission in the DH. Certainly, patch clamp recordings from lamina X neurons have confirmed that block of either GlyT1 or GlyT2 transporters enhances synaptic inhibition by prolonging the decay time course of glycinergic currents (Bradaia *et al*. 2004). Not withstanding these findings, our data indicate that transporter blockade augments tonic glycinergic currents to decrease the activity of PV+ INs, and reduce the inhibition they mediate in DH circuits. Given the clear role PV+ INs have in segregating tactile and nociceptive circuits such a change would allow innocuous tactile input to excite nociceptive circuits and cause allodynia, as has been shown when PV+ INs are selectively ablated (Petitjean *et al*. 2015). This outcome, while seemingly incompatible with the analgesia observed *in vivo*, may be counterbalanced by the concurrent increase in synaptic inhibition arising by other populations of inhibitory interneurons that do not express PV.

In conclusion, this study shows that both synaptic and tonic forms of glycinergic inhibition play critical roles in the normal function of PV+ INs in the spinal DH. As these neurons have been shown to be important in blunting innocuous tactile input to nociceptive circuits, the fast synaptic inhibition provided by glycine receptors may be critical to achieve temporally specific sensory gating in these circuits. Furthermore, tonic glycine currents also provide an additional mechanism for controlling PV+ IN excitability, and consequently, the levels of inhibition they can mediate. Importantly, we also show that heteromeric α/β GlyRs dominate in the generation of both synaptic and tonic currents, an important consideration for future pharmacological targeting of glycinergic inhibition in the DH. Together, these findings suggest that alterations in glycinergic input in the PV+ population could either block or produce pathological sensations such as allodynia. Future work assessing these characteristics in various pain models will determine whether disrupted glycinergic inhibition of PV+ INs contributes to pain under pathological conditions. Furthermore, the capacity of glycine transporter blockade to diminish PV+ IN excitability warrants caution if these approaches are to progress as novel analgesic therapies.

## Additional information

### Competing interests

None declared.

### Author contributions

M.A.G.: conception or design of the work; acquisition or analysis or interpretation of data for the work; drafting the work or revising it critically for important intellectual content. K.A.B.: acquisition or analysis or interpretation of data for the work. R.J.C.: conception or design of the work; drafting the work or revising it critically for important intellectual content. D.I.H.: conception or design of the work. B.A.G.: conception or design of the work; acquisition or analysis or interpretation of data for the work; drafting the work or revising it critically for important intellectual content. All authors have approved the final version of the manuscript and agree to be accountable for all aspects of the work. All persons designated as authors qualify for authorship, and all those who qualify for authorship are listed.

### Funding

This work was funded by the National Health and Medical Research Council (NHMRC) of Australia (grant 631000 and 1043933 to B.A.G.), the BBSRC (grant BB/J000620/1 to D.I.H.), and the Hunter Medical Research Institute (grant to B.A.G. and R.J.C.).
